# Skeletal Muscle MicroRNA and Messenger RNA Profiling in Cofilin-2 Deficient Mice Reveals Cell Cycle Dysregulation Hindering Muscle Regeneration

**DOI:** 10.1371/journal.pone.0123829

**Published:** 2015-04-13

**Authors:** Sarah U. Morton, Mugdha Joshi, Talia Savic, Alan H. Beggs, Pankaj B. Agrawal

**Affiliations:** 1 Division of Newborn Medicine, Department of Medicine, Boston Children's Hospital and Harvard Medical School, Boston, Massachusetts, United States of America; 2 Division of Genetics & Genomics, Department of Medicine, Boston Children's Hospital and Harvard Medical School, Boston, Massachusetts, United States of America; 3 The Manton Center for Orphan Disease Research, Boston Children's Hospital, Boston, Massachusetts, United States of America; University of Louisville School of Medicine, UNITED STATES

## Abstract

Congenital myopathies are rare skeletal muscle diseases presenting in early age with hypotonia and weakness often linked to a genetic defect. Mutations in the gene for cofilin-2 (*CFL2*) have been identified in several families as a cause of congenital myopathy with nemaline bodies and cores. Here we explore the global messenger and microRNA expression patterns in quadriceps muscle samples from cofillin-2-null mice and compare them with sibling-matched wild-type mice to determine the molecular pathways and mechanisms involved. Cell cycle processes are markedly dysregulated, with altered expression of genes involved in mitotic spindle formation, and evidence of loss of cell cycle checkpoint regulation. Importantly, alterations in cell cycle, apoptosis and proliferation pathways are present in both mRNA and miRNA expression patterns. Specifically, p21 transcript levels were increased, and the expression of p21 targets, such as cyclin D and cyclin E, was decreased. We therefore hypothesize that deficiency of cofilin-2 is associated with interruption of the cell cycle at several checkpoints, hindering muscle regeneration. Identification of these pathways is an important step towards developing appropriate therapies against various congenital myopathies.

## Introduction

Congenital myopathies (CMs) are rare muscular disorders characterized by non-progressive or slowly progressive muscle weakness and are usually of genetic origin. Although the genetic basis is now known for many of these conditions, CMs are still sub-classified into various types largely based on pathologic findings.[1,2] Previously, we identified cofilin-2 (*CFL2*) to be mutated in two siblings from a consanguineous family affected by CM with nemaline bodies and minicores.[3] Since then several additional CM patients have been identified carrying *CFL2* mutations.[4,5] Subsequently, we have a created *Cfl2*-knockout mouse model to understand the molecular function of cofilin-2.[6] Cofilin-2-deficient mice are myopathic soon after birth and die by the first week of postnatal life. They initially undergo normal myofibrillar development at birth followed by rapid sarcomeric disruption with nemaline bodies and actin accumulations over the first week of life.

Cofilin-2 is a skeletal muscle-specific member of the AC protein family, which also includes cofilin-1 and destrin. Cofilin-2 is known to interact with actin and tropomyosin, thereby modifying the thin filament.[7] AC proteins are predominantly actin depolymerizers; however, in steady-state assays, cofilin-2 activity has been shown to result in net polymerization, thus likely playing a role in stabilizing the thin filament.[8] Cofilin proteins act in a pH-dependent manner to reorganize the cytoskeleton downstream of Rho/LIM kinase.[9] We have shown that in cofilin-2-deficient mice, embryonic expression of cofilin-1 compensates for cofilin-2 deficiency over the first few days after birth but subsequent muscle maintenance and repair depends on the presence of functional cofilin-2.[6]

Here we further explore the implications of cofillin-2 deficiency by studying messenger RNA (mRNA) and micro RNA (miRNA) expression in skeletal muscles of cofillin-2-deficient mice relative to sibling-matched wild-type mice. Analyzing expression of mRNA and miRNA within quadriceps muscle identified several pathways that are altered due to lack of cofillin-2. These include many aspects of cell cycle regulation and apoptosis. The cell cycle dysregulation may be related to the role of cofilin-2 in regulating mitotic spindle formation, although it is also seen in certain myopathies and dystrophies not linked to cofilin-2.

## Methods

### Animal studies and ethics

All studies were performed with approval from the Institutional Animal Care and Use Committee at Children’s Hospital Boston (Boston, MA, USA) under protocol 13-06-2443R. Generation of *Cfl2*-KO mice was previously described.[6]

### Tissue collection

Six pairs of cofilin-2 deficient and littermate WT mice were euthanized at P7 using inhaled CO_2_ followed by decapitation immediately prior to tissue collection as per the regulations of the Institutional Animal Care and Use Committee at Children’s Hospital Boston. The quadriceps muscles were dissected out and frozen as per standard protocols.

### Messenger RNA array analysis

Separate tissue samples from each of the six cofilin-2 deficient and littermate WT mice were used to generate 12 different datasets. For mRNA analysis, total mRNA was extracted using RNeasy fibrous tissue mini kit from Qiagen (Limberg, Netherlands). RNA quality was analyzed using an Agllent 2100 Bioanalyzer and RNA samples with appropriate size distribution, quantity, and an A260:A280 ratio of 1.8–2.1 were used for analysis. mRNA expression profiles of *Cfl2*-KO and WT pups were obtained by hybridization of the isolated total RNA with GeneChip Mouse Gene 1.0 ST Arrays (Affymetrix, Santa Clara, CA), containing approximately 27 probes for each of the 28,853 genes, and covering the entire mouse transcriptome. Samples were processed by the Microarray Core Facility at Boston Children’s Hospital. The manufacturer's protocols for the GeneChip platform by Affymetrix (Santa Clara, CA) were used. Methods included synthesis of double stranded cDNA from total RNA using reverse transcription reaction, synthesis of cRNA by *in-vitro* transcription, recovery and quantitation of biotin-labeled cRNA, and subsequent hybridization to the microarray slide, post-hybridization washings, and detection of the hybridized cRNAs using a streptavidin-coupled fluorescent dye. Hybridized Affymetrix arrays were scanned with an Affymetrix GeneChip 3000 scanner. Image generation and feature extraction were performed using Affymetrix Genechip Operating Software (GCOS). Raw microarray data were processed by the Affymetrix power tools (APT) software package to generate normalized gene level expression data. Statistical analysis was performed on gene expression data including fold change using geometric means and p-values using Student’s t-test analysis. All data have been uploaded to GEO (ncbi.nlm.nih.gov/geo, (Superseries GSE61451, Subseries 61404).

### MicroRNA array analysis

For miRNA analysis, total RNA was isolated using Ambion miRVana isolation kits (Life Technologies, Carlsbad, CA) and miRNA profiling was performed by LC Biosciences (Houston, TX) on mouse miRBase (Sanger miRBase version 17). Data were normalized and a list of differentially altered miRNAs was created. Most significantly altered microRNAs were defined as those with p values < 0.05. All data have been uploaded to GEO (ncbi.nlm.nih.gov/geo, Superseries GSE61451, Subseries 61449).

### Pathway analysis

Ingenuity Pathway Analysis (IPA) (Ingenuity Systems, Redwood City, CA) version 1.0 software was used to analyze the mRNA and miRNA microarray data. Genes with geometric mean fold change of <0.95 and >1.05, and p-values <0.05 were filtered for analysis. For each individual pathway, p-value of less than 1 E -04 was considered significant. Upstream regulators whose activity could explain the differential expression patterns were also identified by IPA analysis.

### mRNA quantitative real-time polymerase chain reaction (qRT-PCR)

RNA was extracted from quadriceps muscles of P7 cofilin-2 deficient or littermate WT pups using the RNeasy kit (Qiagen) fibrous tissue extraction protocol. One-step qRT-PCR was performed using Superscript III Platinum SYBR Green One-Step qRT-PCR Kit (Invitrogen-Life Technologies, Grand Island, NY), according to manufacturer’s recommendations. Primers were designed based on the GenBank sequences for each target mRNA (see S1 Materials). Control primers for GAPDH were used to normalize for RNA quantity. The reactions were allowed to run on an ABI 7500 real-time PCR machine (Applied Biosystems, Foster City, CA). ΔΔCT and standard deviation values were calculated according to manufacturer instructions.

### miRNA qRT-PCR

miRNA was extracted from quadriceps muscle of P7 cofilin-2 deficient and littermate WT pups using the mirVana miRNA Isolation Kit (Ambien). Quality of samples was assessed using a Bioanalyzer (Agilent). Reverse transcriptions were performed using the microRNA reverse transcription kit (Taqman). qPCR was subsequently perfumed using Taqman primers (see S1 Materials). Control primers for U6 RNA were used to normalize for RNA quantity. The reactions were allowed to run on an ABI 7500 real-time PCR machine (Applied Biosystems, Foster City, CA). ΔΔCT and standard deviation values were calculated according to manufacturer instructions.

### Western blotting

Quadriceps muscles of postnatal day 7 cofilin-2 deficient and wild-type littermate pups were frozen at necropsy and stored at -80°C until analysis. Protein isolation and western blot procedures were performed as described previously.[10] Membranes were probed with antibody against UCP1 (ab23841, 1:500 dilution, Abcam, Cambridge, MA, USA) and GAPDH (FL-335, 1:1000 dilution, Santa Cruz Biotechnology, Santa Cruz, CA, USA) as control. Binding was visualized using SuperSignal West Pico Chemiluminescent Substrate (Thermo Scientific, Rockford, IL) Protein levels were quantified and normalized to GAPDH using the program Quantity One 4.2.1 (Bio-Rad Laboratories, Inc., Hercules, CA, USA) on an Image Station 440 (Kodak DS; Eastman Kodak co., Rochester, NY, USA).

## Results

Gene expression patterns in skeletal muscles from 7-day-old wild type and cofilin-2 deficient mice were analyzed to identify potential mechanisms by which cofilin-2 deficiency results in myopathy. Quadriceps muscle was chosen for array analysis as it is a large proximal lower limb muscle exhibiting typical pathology and often used for microarray analysis. We first looked at the transcripts, both mRNA and miRNA, with the largest magnitude of expression changes. Next, IPA software was used to examine patterns and connections among genes with at least a five-percent change in expression, as described in Methods. Next, pathways and functions that were enriched with those differentially regulated genes were identified. Select mRNA and miRNAs important to our conclusions were verified by qRT-PCR. Each of these analyses indicated significant changes in several pathways, as detailed below.

### Messenger and micro-RNAs with highest fold change

2223 genes were differentially expressed with geometric mean fold change of <0.95 or >1.05, and a p-value of < 0.05 (S1 Data). *Ucp1* (encodes uncoupled protein 1) and *Lep* (encodes leptin), were most downregulated (KO/WT ratio 0.68 and 0.72 respectively) while *Angptl7* (encodes angiopoietin-like 7) and *Rtl1* (encodes retrotransposon-like 1) were both upregulated by ≥30% (KO/WT ratio 1.32 and 1.3 respectively). Forty-nine microRNAs were differentially expressed with p-values of <0.05 (S2 Data). The most significantly downregulated miRNAs were miR-181b and miR-126-3p (KO/WT ratio 0.4 and 0.46 respectively) while most upregulated miRNAs included miR-762 and miR-3960 (KO/WT ratio 4.43 and 4.2 respectively). The ten most upregulated and downregulated genes and microRNAs by p values, and their fold changes are shown in Table 1. Expression changes for 5 miRNAs (let-7b, let-7i, miR-181b, miR-376b, miR-762) were studied by qRT-PCR, and while the expression changes trended in the same direction as the array analysis the results did not achieve statistical significance (S1 Fig).

**Table 1:** The top ten mRNA and miRNAs with largest change in expression.

**DOWNREGULATED**					
**mRNA**	**Ratio KO/WT**	**p-value**	**miRNA**	**Ratio KO/WT**	**p-value**
***Cfl2***	0.48	3.35 E-08	***miR-181b***	0.40	1.43E-04
***Ucp1***	0.68	2.80 E -03	***miR-126-3p***	0.46	5.93E-03
***Lep***	0.72	4.67 E -04	***miR-16***	0.49	3.62E-03
***Glb1l2***	0.75	6.96 E-05	***miR-125a-5p***	0.54	1.93E-02
***Sfrp4***	0.76	1.13 E-05	***miR-23b***	0.55	4.41E-03
***Tmem45b***	0.77	1.66 E-03	***miR-27a***	0.57	2.29E-02
***Grem2***	0.79	2.98 E-07	***miR-23a***	0.57	2.26E-02
***Lrrc38***	0.79	3.36 E-06	***miR-27b***	0.58	2.89E-02
***Cd209f***	0.79	4.07 E-05	***miR-17***	0.59	7.49E-03
***Frzb***	0.79	1.71 E-07	***miR-103***	0.59	9.13E-03
**UPREGULATED**					
**mRNA**	**Ratio KO/WT**	**p-value**	**miRNA**	**Ratio KO/WT**	**p-value**
***Angptl7***	1.32	2.02 E-04	***miR-762***	4.44	3.03E-04
***Rtl1***	1.30	4.40 E-08	***miR-290-5p***	4.20	3.64E-02
***Dsp***	1.27	1.28 E-03	***miR-5105***	3.84	3.43E-02
***Dpep1***	1.25	6.41 E-05	***miR-5130***	3.73	9.85E-03
***Lcn2***	1.25	3.34 E-02	***miR-2137***	3.36	1.94E-03
***Gm10674***	1.25	3.24 E-05	miR-877*	3.29	3.78E-02
***Ankrd1***	1.24	2.08 E-06	miR-328*	3.25	1.31E-02
***Scn3a***	1.24	3.79 E-05	miR-3077*	2.97	4.35E-03
***Serpina3n***	1.22	2.56 E-05	***miR-5109***	2.64	2.05E-02

### Downregulation of cellular growth and proliferation pathways

**Fig 1:**
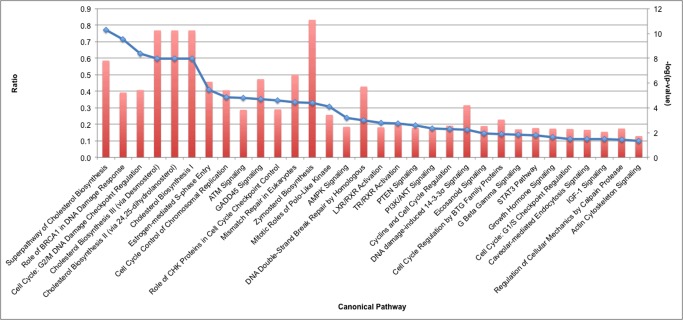
Cellular pathways with differential mRNA expression patterns in quadriceps muscle from *Cfl2* KO mice compared to wild-type littermates. Results were graphed in order of decreasing log(p value), with the dot demonstrating the p-value corresponding to the right-sided y-axis. Ratio refers to the ratio of genes annotated in each pathway that are differentially expressed compared to the total number of genes annotated in the pathway, and is demonstrated by the bar corresponding to the left-sided y-axis.

Pathways with statistically significant enrichment for genes with differential expression included cellular growth and proliferation; cell death and survival; cell cycle; DNA replication, recombination and repair; and cellular assembly and organization (Table 2). Cholesterol biosynthesis, an important pathway indicative of cell growth, was downregulated with the reduced expression of *Srebf1* and *Srebf2*, genes that encode for the sterol regulatory element binding proteins (Fig 1 and S1 Data). Many genes expressed within the pathways of cellular proliferation, viability and survival were generally downregulated. Expression of cell cycle pathway genes during S phase, interphase, and G2 phase was also reduced. For example there was upregulation of *Cdkn1a*, the gene that encodes p21, which causes growth arrest by holding the cell cycle at the G1/S transition. Further, five genes that encode proteins inhibiting mitotic spindle formation, *Fbxo5*, *Kif2c*, *Kif11*, *Sass6* and *Kuf2*, were all upregulated, suggesting a possible reduction in spindle formation. In addition to cell cycle checkpoint changes, several genes involved in DNA damage response, including *Brca1*, were downregulated.

**Table 2:** Cellular functions significantly altered by *Cfl2* deficiency.

Cellular Function	Subpathway	p-value
**Cellular Growth and Proliferation**	Proliferation of cells	1.14 E-22
	Proliferation of muscle cells	1.67 E-09
	Cytostasis	2.37 E-06
	Colony formation of cells	1.74 E-05
	Colony formation	3.09 E-05
	Formation of cells	3.42 E-05
	Arrest in growth of cells	3.89 E-05
**Cell Death and Survival**	Apoptosis	2.73 E-19
	Cell death	1.15 E-18
	Necrosis	6.79 E-18
	Cell death of muscle cells	4.08 E-07
	Necrosis of muscle	4.63 E-07
	Cell viability	9.66 E-07
	Cell survival	1.01 E-06
**Cell Cycle**	Cell cycle progression	6.46 E-17
	Mitosis	9.04 E-15
	Checkpoint control	4.18 E-13
	Segregation of chromosomes	1.59 E-12
	Arrest in mitosis	3.69 E-12
	M phase	4.41 E-12
	Ploidy	2.42 E-10
	Ploidy of cells	1.64 E-09
	Interphase	1.67 E-09
	Cytokinesis	8.97 E-08
	G2 phase	3.13 E-07
	Senescence of cells	4.82 E-07
	DNA recombination	9.85 E-07
	G2/M phase transition	1.52 E-06
	G1/S phase	1.86 E-06
	Polyploidization	2.16 E-06
	Length of mitotic spindle	4.50 E-06
	Polyploidy of cells	5.15 E-06
	Polyploidization of cells	9.06 E-06
	Delay in mitosis	1.03 E-05
	Arrest in G2 phase	1.14 E-05
	S phase checkpoint control	1.21 E-05
	S phase	1.41 E-05
	Arrest in cell cycle progression	2.02 E-05
	Arrest in interphase	2.48 E-05
	Delay in initiation of interphase	3.09 E-05
	Exit from mitosis	3.97 E-05
	Segregation of sister chromatids	3.97 E-05
	Formation of mitotic spindle	4.10 E-05
	Delay in initiation of M phase	4.71 E-05
	G1 phase	6.49 E-05
	Homologous recombination	9.30 E-05
	DNA damage checkpoint	9.40 E-05
	Recombination of cells	9.40 E-05
**DNA Replication, Recombination and Repair**	Checkpoint control	4.18 E-13
	Segregation of chromosomes	1.59 E-12
	Synthesis of DNA	1.65 E-12
	Alignment of chromosomes	2.28 E-09
	Repair of DNA	1.07 E-08
	Morphology of mitotic spindle	2.58 E-08
	DNA damage	1.43 E-07
	Metabolism of DNA	1.57 E-07
	DNA recombination	9.85 E-07
	DNA replication	1.99 E-06
	Condensation of chromosomes	2.16 E-06
	Recombination	5.46 E-06
	Chromosal congression of chromosomes	6.10 E-06
	Abnormal morphology of mitotic spindle	1.10 E-05
	Quantity of mitotic spindle	1.10 E-05
	S phase checkpoint control	1.21 E-05
	Damage of chromosomes	1.41 E-05
	Homologous recombination repair of DNA	1.59 E-05
	Breakage of chromosomes	2.12 E-05
	Segregation of sister chromatids	3.97 E-05
	Double-stranded DNA break repair	4.08 E-05
	Formation of mitotic spindle	4.10 E-05
	Formation of nuclear foci	7.26 E-05
	Homologous recombination	9.30 E-05
	DNA damage checkpoint	9.40 E-05
	Recombination of cells	9.40 E-05
**Cellular Assembly and Organization**	Segregation of chromosomes	1.59 E-12
	Alignment of chromosomes	2.28 E-09
	Missegregation of chromosomes	1.17 E-06
	Organization of cytoskeleton	3.61 E-06
	Attachment of spindle fibers	6.10 E-06
	Chromosomal congression of chromosomes	6.10 E-06
	Organization of cytoplasm	8.27 E-06
	Quantity of mitotic spindle	1.10 E-05
	Polymerization of filaments	1.57 E-05
	Polymerization of microtubules	2.54 E-05
	Segregation of sister chromatids	3.97 E-05
	Formation of mitotic spindle	4.10 E -05
	Quantity of chromosome components	6.75 E-05
	Formation of nuclear foci	7.26 E-05

**Fig 2:**
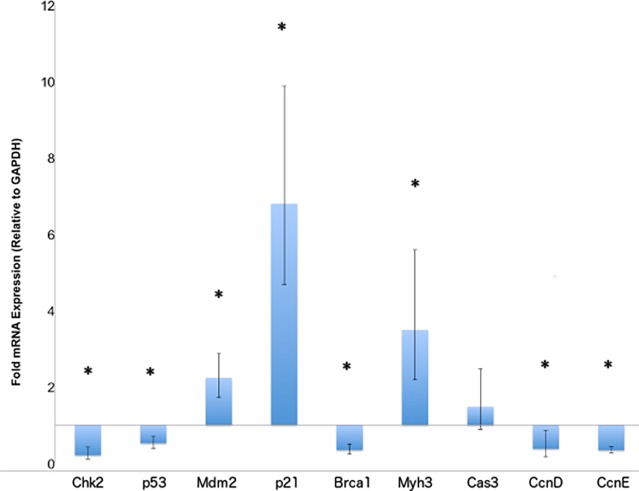
qRT-PCR confirmation of gene expression changes related to cell cycle regulation. qRT-PCR was performed on mRNA isolated from quadriceps muscle of *Cfl2* KO mice and compared to wild-type littermates. GAPDH was used as a control. Fold changes are plotted, with standard deviation indicated by error bars. Asterisks indicate significant differences (p-value <0.05) between groups.

To confirm alterations in the levels of mRNA transcripts involved in cell cycle regulation and muscle repair, we utilized qRT-PCR experiments. A significant increase in the *Cdkn1a* (p21) transcript levels was confirmed in the *Cfl2*-KO mice, while downstream targets inhibited by p21 including *Ccnd* (cyclin D), *Ccne* (cyclin E) were significantly reduced as was seen in the microarray data (Fig 2 and S1 Data). We looked at the p21 upstream regulators, including *Mdm2* (upregulated) and *Chek2* (downregulated), both present in the 2223 genes list. As with microarray data, *Mdm2* was upregulated and *Chek2* encoding for CHK2 downregulated on qRT-PCR experiments (Fig 2). We also evaluated Trp53 (encoding p53) levels using qRT-PCR, not present in the 2223 gene list but downregulated on microarray data with a p value <0.005. The p53 levels were significantly reduced on qRT-PCR (Fig 2). We hypothesize that an increase in the levels of *Mdm2* caused a reduction in both *Chk2* and *p53* transcripts.[11,12]. *Myh3* (which encodes myosin heavy chain 3), is known to be upregulated in muscle satellite cells undergoing repair, and was confirmed to be upregulated in qRT-PCR experiments as well (Fig 2).

### Altered actin cytoskeleton, apoptotic and mitotic pathways

**Fig 3:**
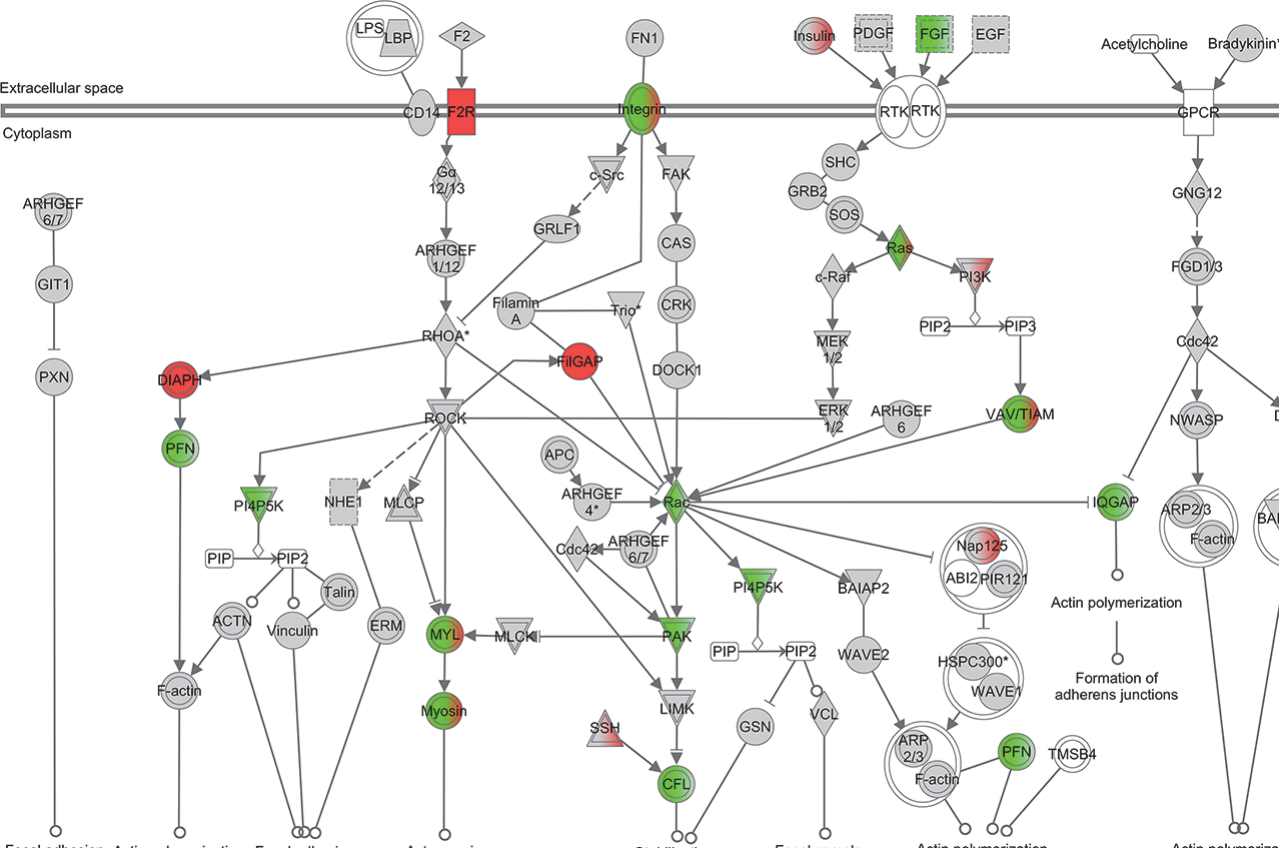
Differential mRNA expression in quadriceps muscle from *Cfl2* deficient mice compared to wild-type littermates for actin cytoskeleton. Red indicates increased expression and green indicates decreased expression. The brighter the red or green color, the more significantly altered is the expression. When both colors are present for the same gene, it indicates that some isoforms of the gene are upregulated while others are downregulated. White or gray boxes indicate no significant differential expression.

**Fig 4:**
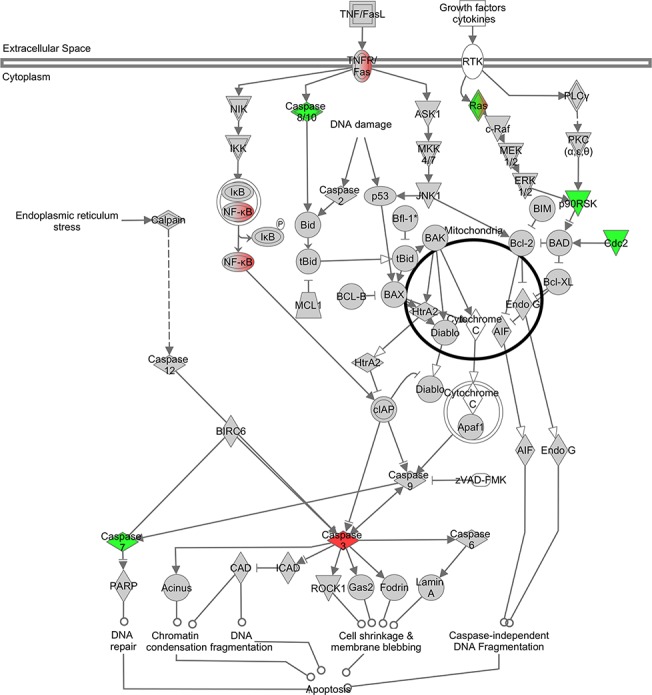
Differential mRNA expression in quadriceps muscle from *Cfl2* deficient mice compared to wild-type littermates for apoptosis. Color representations are the same as described in Fig 3.

**Fig 5:**
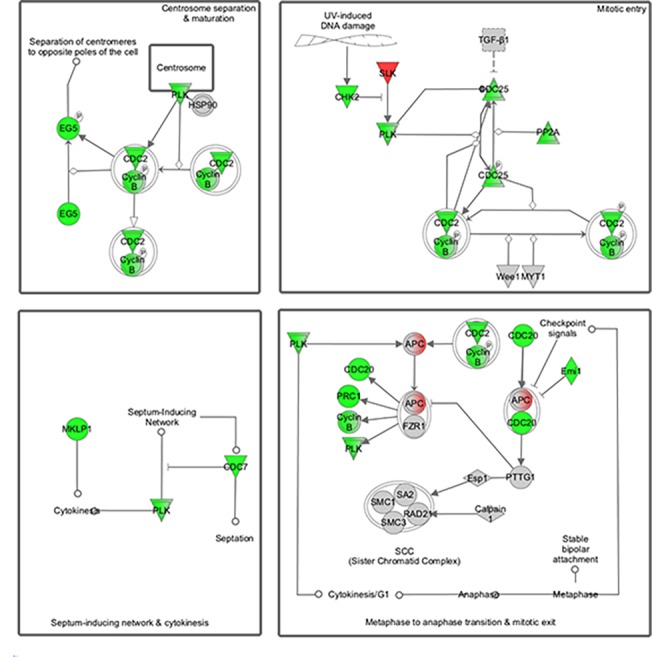
Differential mRNA expression in quadriceps muscle from *Cfl2* deficient mice compared to wild-type littermates for cell cycle. Color representations are the same as described in Fig 3.

**Fig 6:**
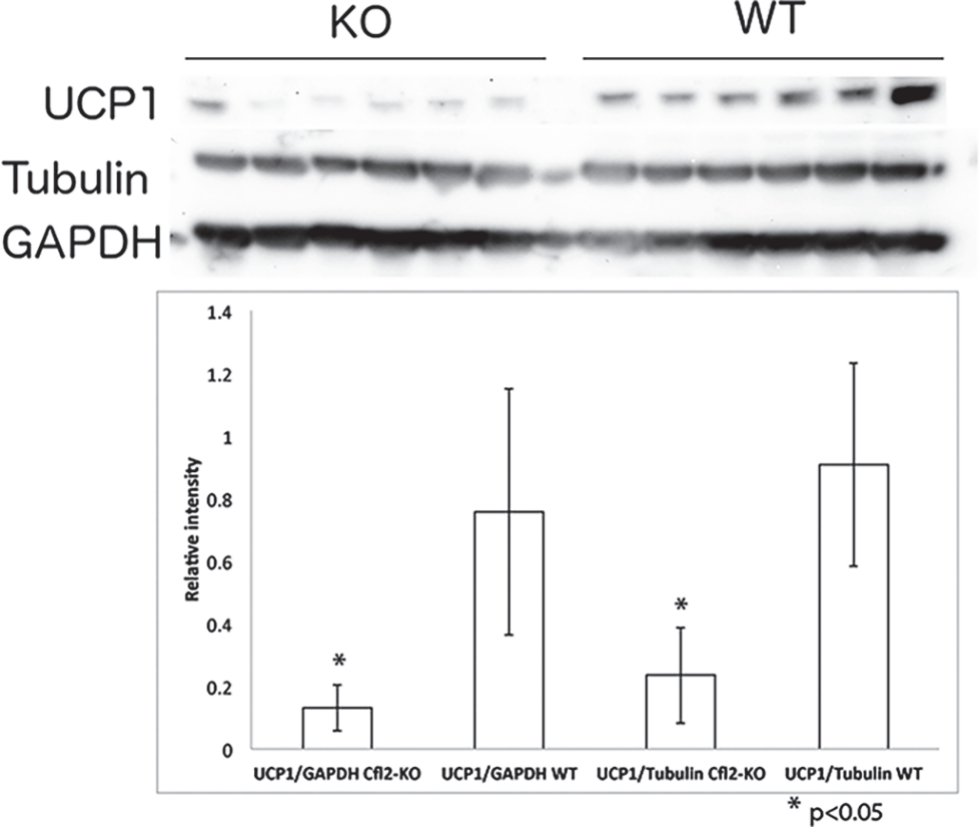
Expression of UCP1 is decreased in *Cfl2* deficient mice. Western blot for UCP1 protein demonstrated decreased expression of UCP1 in 6 individual *Cfl2* deficient mice (KO) compared to 6 individual wild-type littermates (WT). Western blot for Tubulin and GAPDH did not demonstrate any differences in expression, as a control for protein loading. Densitometry was used to demonstrate this quantitatively, with statistically significant decreases of UCP1 seen in *cofilin*-2 deficient mice compared to wild-type littermates when normalized to either GAPDH (left bars) or Tubulin (right bars). Asterisks indicate the p-value for difference between KO and WT mice was <0.05 for both comparisons.

Pathways with statistically significant changes were evaluated in more detail. Among genes involved in the actin cytoskeleton pathway, there was a significant increase in expression of *Ssh2*, which encodes a slingshot phosphatase responsible for cofilin dephosphorylation. Meanwhile, genes encoding for proteins upstream of Lim kinase that phosphorylate cofilin, including *Fgf18* (FGF), *Pak1* (PAK) and *Rac3* (RAC), were all downregulated (Fig 3). The apoptotic signaling pathway was significantly altered with increased expression of *Casp3* and *Fas* and reduction in *Casp7 and Casp8* (Fig 4). Those are the same genes identified as being significantly altered in the pathways related to muscle necrosis and cell death in Table 2. qRT-PCR for *Casp3* showed upregulation as seen with microarray, but did not reach statistical significance (Fig 2). Given the increase in genes inhibiting mitotic spindle formation, we reviewed mitosis in greater depth. A significant downregulation of the majority of genes involved in mitosis was noted (Fig 5). Finally, to assess changes in *Ucp1* expression that could be related to mitochondrial membrane potential and apoptotic threshold, we examined UCP1 protein levels. Western blot analysis of muscle samples from the 6 pairs of *Cfl2*-KO and wild type mice found a marked reduction in UCP1 levels in the cofilin-2 deficient mice, consistent with downregulation of *UCP1* transcript (Fig 6).

### MicroRNA expression patterns also predicted to target cell cycle pathways

miRNA expression patterns were analyzed to identify biological pathways whose genes were enriched among known targets of miRNAs in the dataset. Similarly to the mRNA expression arrays, significant enrichment was predicted for transcripts involved in cell cycle control; cellular growth and proliferation; and cell death and survival (Table 3). Of note, changes in miRNA known to regulate DNA replication and repair were present in the mRNA data but not present in the miRNA data.

**Table 3:** Cellular functions significantly altered by *Cfl2* deficiency.

Cellular Function	Subpathway	p-value
**Cell Cycle**	G1/S phase transition of fibroblast cell lines	6.63 E-07
**Cellular Development**	Proliferation	2.78 E-06
**Cellular Growth and Proliferation**	Proliferation	2.78 E-06
**Cell Death and Survival**	Apoptosis	6.29 E-05

## Discussion

Here we report an integrated analysis of mRNA and miRNA expression profiles in quadriceps muscles from a mouse model of CM. Changes in both mRNA and miRNA expression indicate that alterations in expression of genes responsible for cell cycle inhibition is a key finding in cofilin-2 deficiency. In the setting of myopathy, muscle regeneration is needed, but the profiling data here points to a lack of successful regeneration. Indeed, pathological studies have shown decreased regeneration in muscle from patients with NM, despite increased progenitor satellite cell populations.[13] This could lead to exhaustion of satellite cell regenerative capacity, as is observed in Duchenne muscular dystrophy (DMD).[14] Such impaired regeneration could underpin the progressive muscle pathology seen in NM.

### Impaired Cell Cycle Regulation: Regenerative Block?

There is precedent for cell cycle dysregulation as a consequence of muscle disease. A comparison of gene expression patterns in patients with DMD and aging skeletal muscle identified cell cycle dysregulation in addition to fibrosis.[15] Increase in *p21* mRNA levels has been previously demonstrated in fibroblasts from a patient with severe CM.[16] p21 activation is also correlated with the terminal cell cycle arrest of myocytes.[17] Dux4 overexpression, as is seen in facioscapulohumeral dystrophy, leads to increased p21.[18] This was mediated by Dux4 recruitment of Sp1 to the promoter region of p21. Conversely, p21 is epigenetically silenced in young muscle stem cells compared to old, both in quiescent cells and those responding to injury, and ectopic FGF-2 signaling in aged muscle can silence p21 and restore cell proliferation.[19] In a model of myositis, p21 levels were decreased, prompting cell cycle reentry.[20] Similarly, in rhabdomyoscarcoma TBX2 inhibits p21 via HDAC1 recruitment, thereby promoting cell proliferation.[21] Inhibition of p21 in primary myoblasts from patients with Duchenne muscular dystrophy improved cellular proliferation.[22] Overall, reducing p21 levels may be a potential therapeutic option against CMs.

However, some p21 is necessary for muscle repair as myoprogenitor cells from p21 knockout mice display increased apoptosis and a marked impairment in their ability to differentiate.[23]

This blockade of the cell cycle could explain the lack of successful muscle regeneration despite activation of satellite cell populations and expression of regenerative genes including *Myh3* and *Myh8*, similar to DMD studies.[13,24] Previous NM expression studies have specifically identified evidence of decreased muscle proliferation associated with slow fiber predominance.[25] *Itga5* and *Vcl* reach peak expression levels during cell-cycle withdrawal and were both upregulated in our model of CM consistent with cell cycle withdrawal due to cofilin-2 deficiency.[26] This blockade in muscle regeneration would cause the accumulation of satellite cells as previously described in NM.[13]

### Increased Apoptosis

Another mechanism by which proliferative signals could be blocked is via increased cell death. In addition to cell cycle dysregulation, there is also increased expression of several pro-apoptotic genes within the muscles of affected mice. Specifically, there are significant increases in caspase-3 and FAS. Increased apoptosis has been noted previously in studies of congenital myopathies, and in mouse models the inhibition of apoptosis has led to improvement in the muscle pathology and functional outcomes.[27,28] Compared to previous studies [29], however, we see no sign of increased calpain expression triggering apoptosis.

Changes in apoptosis are often linked to changes in the mitochondrial membrane potential. The most significantly altered gene expression due to cofillin-2 deficiency was *Ucp1*, downregulated by 30%. *Ucp1* encodes a mitochondrial protein that has a role in brown fat metabolism. Many studies in the past have found gene expression changes implicating mitochondrial dysfunction in NM, including studies that have found *Ucp3* expression changes to be a marker of NM compared to other myopathies.[30] Interestingly, our previous study identified significant increase in UCP3 protein expression despite downregulation of the *Ucp3* mRNA in NM patients.[13]

### Cholesterol and Myopathy

In humans it has been documented that adequate serum cholesterol is correlated with the ability to induce muscle hypertrophy through strength training.[31] Further, the composition of lipid rafts in myoblasts changes during the process of differentiation. Modulation of cholesterol availability can alter muscle differentiation via phosphorylation of mTORC1, thereby affecting regenerative capacity.[32] Interestingly, another study in chick embryos demonstrated that cholesterol depletion can bypass the anti-mitotic effects of cytosine arabinoside. Thus the inhibition of cholesterol biosynthesis observed in cofilin-2 deficiency may be compensatory rather than a primary result of the mutation.[33] Given the ability to modify cholesterol amounts through diet and medications, further investigation of this aspect of NM is warranted.

### Regulation of actin filaments

Within our model of cofilin-2 deficiency, *Ssh*, encoding for slingshot phosphatase, a protein that dephosphorylates cofilin (active form), was upregulated. This would lead to increased activity of the residual cofilins that are present.[34] Conversely, there was a decrease in Fgf/Pak/Rac activity leading to reduction in Lim kinase activity, which in turn lowers cofilin phosphorylation thereby decreasing levels of the active form.[35] An increase in the activity of remaining cofilins may be one mechanism by which myofibers attempt to compensate for the lack of cofilin-2.

### Putative Mechanisms

We hypothesize that deficiency of cofilin-2 protein leads to a block in the cell cycle that renders skeletal muscle unable to repair, due to blockade in the differentiation of satellite cells. Thus the increased regenerative signals, evidenced by upregulation of *Myh3* and *Myh8* expression, are inhibited by increased p21 activity leading to cell cycle blockade. If p21 is in fact preventing regeneration, modulation of the p21 pathway could have therapeutic benefit to patients. Further experiments, and comparison with expression arrays from other mutations that lead to NM, will help to elucidate the relationship between cofilin-2 and the changes in cell cycle gene expression. These results raise several non-mutually exclusive mechanisms by which cofilin-2 deficiency may cause the rapidly progressive myopathy seen in the mouse knockout model. In summary, our expression data from cofilin-2 knockout mice suggests that the muscular dysfunction in human patients carrying *CFL2* mutations may be due to a combination of decreased regenerative repair, increased apoptosis and mitochondrial dysfunction, and therapies targeting those pathways may be good candidates to help ameliorate the disease.

## Supporting Information

S1 Fig qRT-PCR confirmation of miRNA expression changes related to cell cycle regulation. qRT-PCR was performed on miRNA isolated from quadriceps muscle of *Cfl2* KO mice and compared to wild-type littermates. U6 RNA was used as a control. Fold changes are plotted, with standard deviation indicated by error bars. There were no significant differences (p-value <0.05) between groups.
(TIF)

S1 Data 2223 mRNA genes included in analysis. (XLSX)

S2 Data 49 miRNA genes included in analysis. (XLS)

S1 Materials qRT-PCR primer sequences. (DOCX)
