# Supplementary figures and images for: Skeletal Muscle MicroRNA and Messenger RNA Profiling in Cofilin-2 Deficient Mice Reveals Cell Cycle Dysregulation Hindering Muscle Regeneration
Source: PLoS One. 2015 Apr 13;10(4):e0123829. doi: 10.1371/journal.pone.0123829 (PMC4395318; doi:10.1371/journal.pone.0123829)

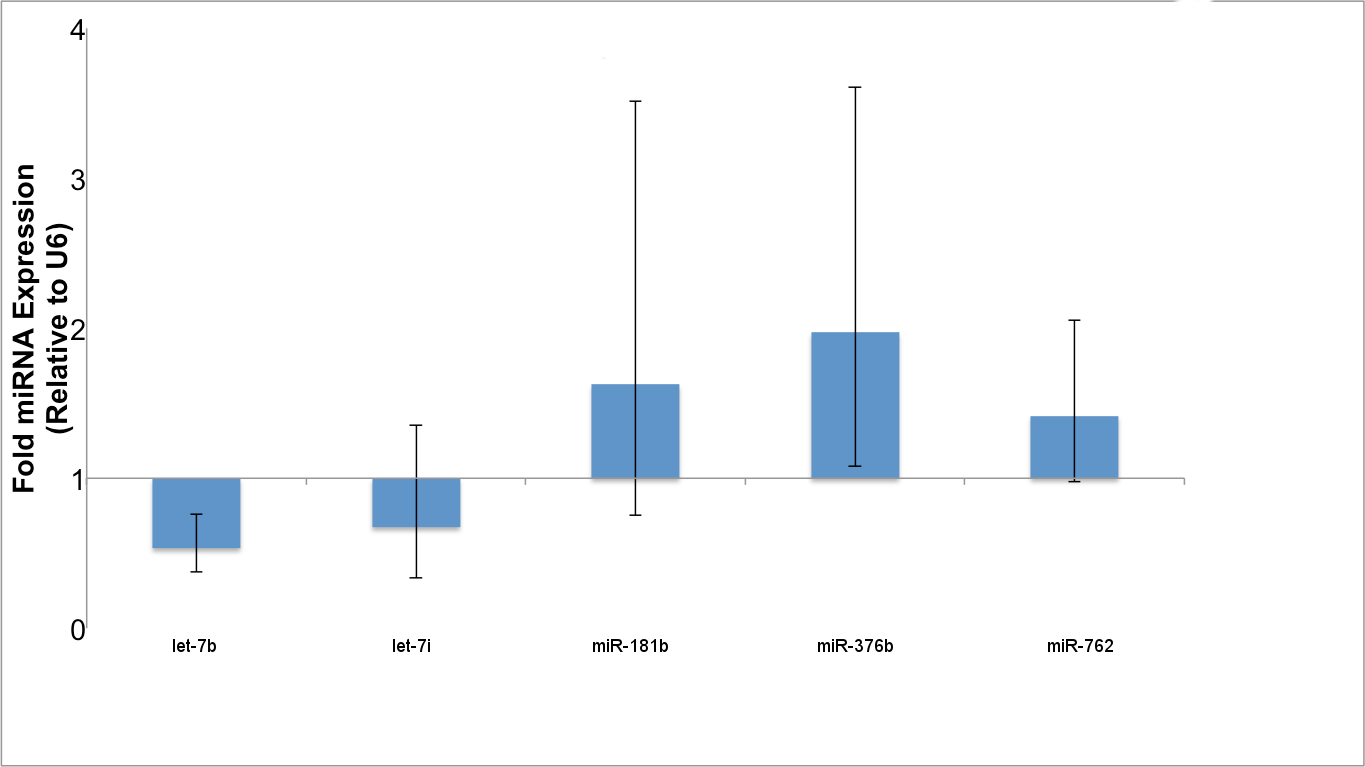

Supplement: S1 Fig — qRT-PCR was performed on miRNA isolated from quadriceps muscle of Cfl2 KO mice and compared to wild-type littermates. U6 RNA was used as a control. Fold changes are plotted, with standard deviation indicated by error bars. There were no significant differences (p-value <0.05) between groups. (TIF) [file pone.0123829.s001.tif]
