# Supplementary material for: Skeletal Muscle MicroRNA and Messenger RNA Profiling in Cofilin-2 Deficient Mice Reveals Cell Cycle Dysregulation Hindering Muscle Regeneration
Source: PLoS One. 2015 Apr 13;10(4):e0123829. doi: 10.1371/journal.pone.0123829 (PMC4395318; doi:10.1371/journal.pone.0123829)
Supplement: S1 Materials — (DOCX) [file pone.0123829.s004.docx]

**Primers sequences used for SYBR green QRT-PCRs**

mRNA_BRCA1_tran1_7_F ACACGCGCTTAACCTCAGTC

mRNA_BRCA1_tran1_7_R TCTTCCTGTCCCTGACGTTC

mRNA_TBX2_re_F AGAGGTGCAGCAAGGAACC

mRNA_TBX2_re_R CACCGAGGCTAGCAGATGAC

mRNA_Caspase3_re_F TTCCTGGAGAAATTCAAAGGAC

mRNA_Caspase3_re_R CAGTTCTTTCGTGAGCATGG

new_MDM2_F TGTTTGGAGTCCCGAGTTTC

new_MDM2_R ATCCTGATCCAGGCAATCAC

CHK2_F CCGGACTTACAGCAAGAAGC

CHK2_F CCGGACTTACAGCAAGAAGC

mRNA_CDKN1A_F GTCCAATCCTGGTGATGTCC

mRNA_CDKN1A_R CAGGGCAGAGGAAGTACTGG

mRNA_MYH3_F ACCGTGAGGAACGACAACTC

mRNA_MYH3_R ATCACAGCCCCTGTCAGTTT

mRNA_MYH8_F ACCGTCAACCCCTACAAGTG

mRNA_MYH8_R CGTGGTACCGAAGTGGATTC

mRNA_P53_F CGGGTGGAAGGAAATTTGTA

mRNA_P53_R CTTCTGTACGGCGGTCTCTC

**Taqman probes for miRNA QRT-PCRs :**

1. has_let_7b TM: 002619
2. has_let_7i TM: 002221
3. mmu_miR_376b TM: 002452
4. mmu_miR_762 TM: 002028
5. mmu_miR_181b TM: 465209
6. u6 SnRNA TM: 1973
